# From policy to practice: premarital spinal muscular atrophy screening as a public health initiative in northern Türkiye

**DOI:** 10.3389/fpubh.2025.1714795

**Published:** 2026-01-21

**Authors:** Feyza Nur Topcu Yenercag, Sule Ozturk, Gunay Kaya Tarhan

**Affiliations:** 1 Department of Public Health, Faculty of Medicine, Samsun University, Samsun, Türkiye; 2 Samsun Provincial Health Directorate, Samsun, Türkiye

**Keywords:** carrier screening, epidemiology, genetic counseling, premarital screening, spinal muscular atrophy (SMA)

## Abstract

**Background:**

Spinal muscular atrophy (SMA) is a severe autosomal recessive neuromuscular disease and a major cause of infant mortality. Türkiye implemented a nationwide premarital SMA carrier screening program in 2021 to reduce disease incidence through early detection and genetic counseling.

**Methods:**

This study evaluates the application of the program in a northern province of Türkiye, covering 19,988 individuals screened between December 27, 2021, and May 31, 2024.

**Results:**

The carrier prevalence was 2.26% (1:44), and the screening uptake rate was 87.9%. Of 441 screened partners of identified carriers, 13 were also found to be carriers. Seven couples were confirmed as dual carriers and were referred for preimplantation genetic diagnosis (PGD), effectively preventing the birth of affected children.

**Conclusion:**

The findings demonstrate the value of premarital screening in populations with high consanguinity rates. The study supports the integration of accessible, socially informed screening practices and highlights the need for further data collection, expanded carrier panels, and enhanced public awareness in Türkiye.

## 1 Introduction

Spinal muscular atrophy (SMA) is a neuromuscular disease that is one of the most frequent genetic causes of infant mortality worldwide. Additionally, it is also the second most common and deadly autosomal recessive disease (1). SMA frequently occurs with homozygotic deletion or mutation of the survival motor neuron (SMN) gene located on the 5th chromosome. If the SMN protein cannot be produced at adequate levels, alpha motor neuron degeneration occurs, and muscle atrophy develops (2). The most frequent mutation occurring on the SMN1 gene is the deletion of exon-7 located on the 5th chromosome (3).

SMA is frequently diagnosed late after symptoms appear, as it is a rare disease with heterogeneous findings. According to a systematic review, the global incidence of SMA is 10 per 100,000 live births. The prevalence was determined to be 1–3 per 100,000 people, with the low detection thought to be largely due to the shortened life expectancy (1). According to a multinational study investigating different ethnic groups, the SMA carrier frequency ranges from 1:47 to 1:72 (4).

**Figure 1:**
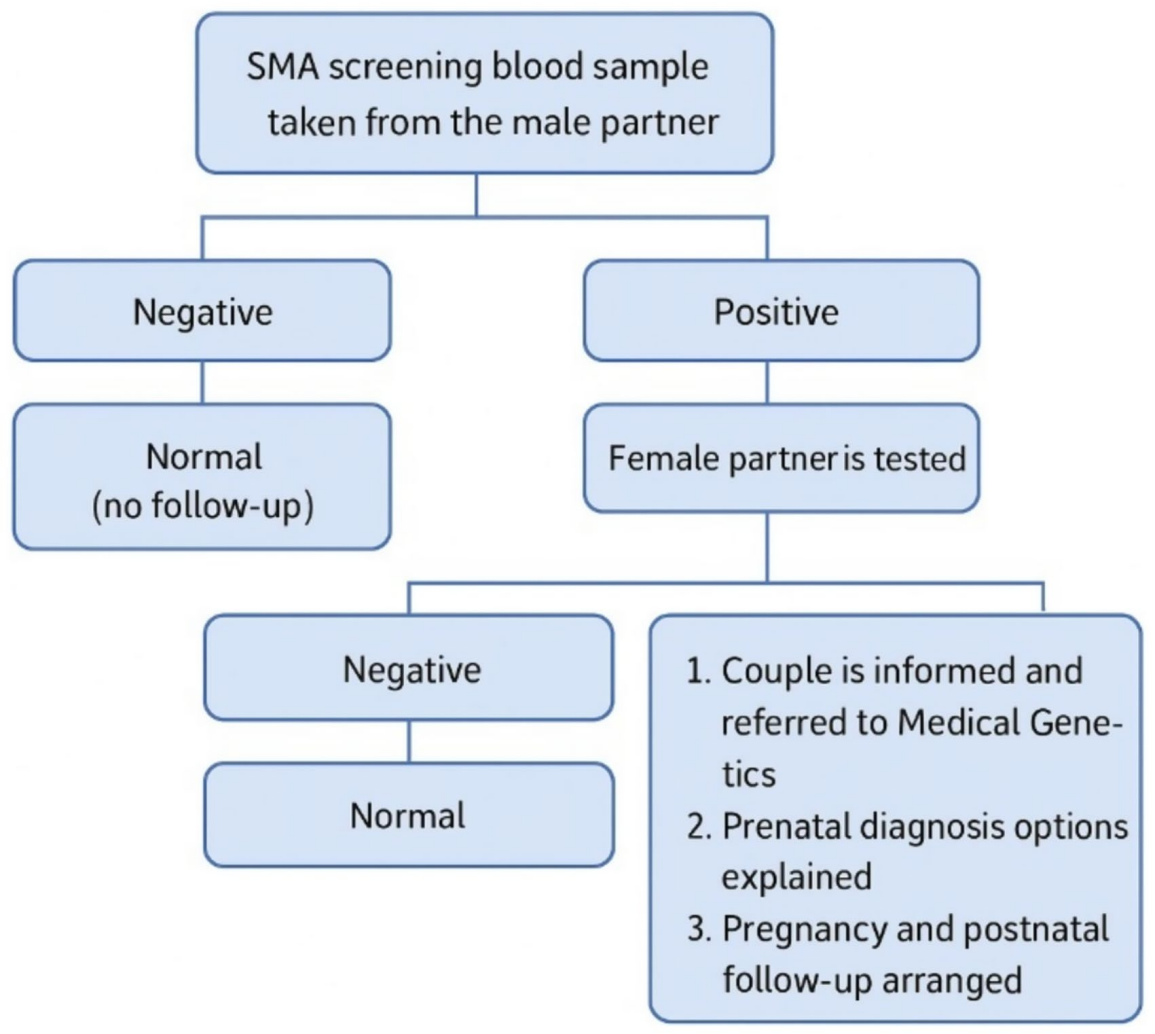
National premarital SMA carrier screening program algorithm – Türkiye (5). The residual risk for those testing “Negative” due to the false negativity of qPCR should be considered.

In Türkiye, the incidence of SMA and carrier rates are not clearly known. However, it is thought that our country, with frequent consanguineous marriage, has high rates of autosomal recessive heritable diseases and carrier rates. Considering that nearly 1,100,000 live births have occurred per year in recent years, the annual new case number is predicted to be between 130 and 180 (mean: 150). Nearly 3,000 SMA patients are monitored (Figure 1) (5).

SMA is divided into four subtypes according to age at onset and severity of muscle weakness. SMA type 1 (Werdnig-Hoffman disease) is the earliest and most severe form, causing severe and progressive respiratory problems and hypotonia in the first 6 months of life. SMA type 2 (subacute form) generally begins from 6 to 18 months with symptoms similar to type 1, though the symptoms do not worsen rapidly. SMA type 3 (Kugelberg-Welander disease) begins after the age of 2 years, and the first symptom is difficulty walking. Type 4 begins in adulthood and is the mildest form.

Potential treatment approaches were developed with the understanding that SMA disease has molecular genetic features (6). Two types of treatments (nusinersen and zolgensma) received FDA approval and significantly improved long-term quality of life (7). Therapeutic approaches are known to increase the level of SMN in motor neurons when applied presymptomatically. Thus, early diagnosis and treatment can slow the neurodegenerative process (8).

For genetic diseases, primary (screening of society for carriers, screening of populations at risk, premarital screening programs, preconception screening), secondary (newborn screening (NBS), treatment of presymptomatic infants), and tertiary (treatment of symptomatic infants) protection is available. Due to NSP, SMA disease is detected in the early period, and it is known that severe forms may be prevented with presymptomatic treatment. Health economic studies revealed the value of NSP. In a study presenting the results of 2 years of SMA NBS in Türkiye, asymptomatic infants who received early nusinersen treatment achieved timely acquisition of the World Health Organization motor developmental milestones (9). However, in upper middle-income countries, a patient burden greater than the system can treat may occur (10). This increases the importance of premarital screening to prevent the birth of sick infants (11). A premarital SMA screening program is implemented throughout Türkiye. The aim of the program is to offer genetic counseling and prenatal or preimplantation diagnostic test options to couples identified as carriers. Additionally, the long-term target is to reduce morbidity and mortality linked to SMA (5).

According to the Premarital SMA Carrier Screening Guidelines, given the high carrier rates in Türkiye, SMA carrier screening is recommended for all couples prior to pregnancy. If both partners are identified as carriers, the program aims to support healthy offspring through genetic counseling and options such as prenatal or pre-implantation diagnostic testing. Within this framework, the premarital SMA carrier screening program was launched in Türkiye as of December 2021. In practice, blood samples are first collected from male partners, and those identified as carriers are followed up for confirmation and genetic counseling. Additionally, healthcare personnel provide information to couples, carriers are registered, pregnancy monitoring is conducted, and births of affected children are tracked. The program was implemented in 2022, and its coverage has steadily increased as training for healthcare professionals and family physicians, along with public awareness campaigns, has been conducted.

This study aims to evaluate the implementation and effectiveness of the national premarital SMA carrier screening program conducted over the past 3 years in a province in northern Türkiye, based on participation rates and carrier prevalence.

## 2 Methods

This was an epidemiological and cross-sectional study. The SMA screening results obtained between 27 December 2021 and 31 May 2024 were retrospectively assessed. The study received permission from the Clinical Research Ethics Committee.

### 2.1 Study population

In all provinces, the Premarital National SMA Carrier Screening Program was begun by the Republic of Türkiye Ministry of Health in December 2021. In this study, the screening results for 19,988 people from 27 December 2021 to 31 May 2024 in Samsun, Türkiye were evaluated. Samsun is the largest province in the northern region of Türkiye and comprises17 districts, including four central ones. The provincial population is 1,377,546. More than half of this population lives in the central counties. All participants who applied to their family clinician for a premarital health report or carrier screening and provided written informed consent were included in this study.

In Türkiye, family practitioners provive participants with information about SMA and its screening. Screening involves a three-stage screening procedure. In the first stage, blood samples are collected for screening from the male partner/spouse candidate. Blood samples of 2–3 cc are taken in an EDTA tube, coded with a barcode and entered into the system. If the tubes are not sent to the laboratory on the same day, they are stored at +4 °C in a refrigerator. The tubes are sent to the General Directorate of Public Health Microbiology Reference Laboratories (two in Ankara and İstanbul) after being placed in suitable boxes to prevent breakage and are tested there. If the result is an “SMA carrier”, the female partner/spouse candidate is screened in the second stage. If a male partner declines the test, the family physician informs him about the importance of the disease and the possible consequences. Then, the test may be performed on the female partner. If the couple refuses the test despite being informed, a document is issued to record this situation. The screening results are notified to family clinicians through the system, and people may view their results through the Turkish Ministry of Health integrated national personal health record system. If both partners/spouse candidates are carriers, couples are referred to a medical genetic expert in the last stage. They are informed about detailed genetic counseling services, risks, and prenatal diagnostic possibilities and referred to preimplantation genetic diagnosis centers accordingly. According to the guideline, genetic counseling mainly focuses on risk assessment, carrier screening, and reproductive options. The possibilities for prenatal diagnosis and the options for having a healthy child are also explained. In addition, genetic counseling provides information about possible SMA treatment in case of positive prenatal testing.

### 2.2 Quantitative real-time PCR analysis

The Premarital National SMA Carrier Screening Program uses the real-time polymerase chain reaction (real-time PCR/RT-PCR) method. Real-time quantitative PCR is a simple, rapid, and cost-effective method for large-scale routine SMA carrier screening (12). It is one of the molecular genetic methods with at least 95% sensitivity and specificity for showing copy number changes in the exon 7 and exon 8 regions, especially, on the SMN1 gene. Linked to the method, deletion of the SMN1 gene may be missed at rate of 5%, leading to false-negative results. Most SMA carriers (95%) have biallelic deletions in exon 7 of the SMN1 gene, while the remaining 5% carry various other point mutations that are not detectable through standard carrier screening. Additionally, approximately 4% of the population possesses varying SMN1 copy numbers, which can lead to inaccurate results in carrier screening tests (13). Those who tested positive in the screening were defined as carriers, while individuals confirmed by MLPA, the gold standard diagnostic method, were defined as confirmed carriers. The rate of the follow-up compliance of partners was identified as “recall rate.”

### 2.3 Data gathering

Information like screening time, age, sex, and address of people participating in the SMA carrier screening was obtained from the Laboratory Information Management System (LIMS). The number of marriages was obtained annually from the Turkish Statistical Institute (TÜİK).

### 2.4 Ethics approval and consent to participate

The investigation was carried out in compliance with relevant laws, guidelines, and the ethical standards of the Declaration of Helsinki. The study was approved by the Samsun University Clinical Ethics Board (approval number: 2024/11/8, date: 05.06.2024). All participants provided written informed consent. All participants provided consent for publication.

### 2.5 Statistical analysis

Statistical analysis in the study was performed using SPSS (IBM Statistical Packages for the Social Sciences; Armonk, NY, United States) Version 20.0. The information is presented in Tables 1, 2. Data that did not fit a normal distribution were expressed as the median (minimum-maximum). Chi-square was used to analyze categorical variables. The value for statistical significance was accepted as *p* < 0.05. Categorical data were expressed as numbers (*n*) and percentages (%). Because there were no missing data, all analyses proceeded without the need for imputation.

**Table 1:** Premarital National SMA Carrier Screening Program results.

Variable	2022	2023	2024 (5 months)	*p* value*	Total
Number of marriages, *n*	9,140	9,058	–		–
Number of people screened, *n*	7,306	8,698	3,984		19,988
Uptake rate, %	79.9	96.0	–	**<0.001**	–
95% CI, %	79.1–80.7	95.6–96.4	–		–
Number of carriers (PCR), *n*	160	217	76		453
Carrier proportion, %	2.19	2.49	1.91	0.102	2.27
95% CI, %	1.85–2.53	2.16–2.82	1.48–2.34		2.06–2.48
Partners, *n*	156	212	73		441
Recall rate, %	97.5	97.7	96.0	0.737	97.4
95% CI, %	95.1–99.9	95.7–99.7	91.6–100.4		95.9–98.9
Carrier couples, *n*	2	3	2		7
Carrier rate of partners, %	1.28	1.42	2.74	0.686	1.59
Preimplantation genetic diagnosis, *n*	1	2	0		3

*Chi-Square test. Bold *p*-values indicate statistical significance (*p* ≤ 0.05).

**Table 2:** Features of 453 carriers detected in Samsun province within the scope of the Premarital National SMA Carrier Screening Program.

Demographic features			
Age (years, median (min-max))		29.0 (19.0–69.0)	
	*n*	%	95% CI
Age, years			
<25	98	21.6	17.8–25.4
26–34	284	62.7	58.2–67.2
>35	71	15.7	12.3–19.1
Residence			
Central counties	194	42.8	38.2–47.4
Other counties	181	40.0	35.5–44.5
Outside of province	78	17.2	13.7–20.7

## 3 Results

A total of 19,988 people participated in the premarital national SMA carrier screening (Table 1). In 2022, there were 9,140 marriages, with 7,306 people participating in screening. In 2022, the uptake rate was determined to be 79.9%. In 2023, the number of marriages was 9,058. In 2023, 8,698 people participated in screening, with a 96.0% uptake rate. qPCR was used as a screening method. Multiplex ligation-dependent probe amplification (MLPA) was used for carrier confirmation. Of the 19,988 people tested, 453 were SMA carriers. This shows that the prevalence of being a carrier was 1:44 (2.26%). Screening coverage differed significantly between 2022 (79.9%) and 2023 (96.0%) (*p* < 0.001). However, carrier prevalence did not differ significantly across years 2022 (2.19%), 2023 (2.49%), and 2024 (1.91%) (*p* = 0.102). Similarly, recall rates (*p* = 0.737) and carrier couple rates (*p* = 0.686) showed no significant differences across the 3 years. The analysis for PGT eligibility also showed no significant variation (*p* = 0.327).

Table 2 shows the features of the 453 carriers. Of these, 284 (62.7%) were 25–35 years of age, and 194 (42.8%) were living in the central counties. The 453 healthy SMA carriers with heterozygous deletion in exon 7 of SMN1 were informed about the mode of inheritance and reproduction risk of the disease. They were referred to a genetic clinic to confirm carrier status with the MLPA method and detailed genetic counseling. The spouse candidates for the 453 carriers who were informed about the disease were called and screened with qPCR. Despite being informed, 12 spouse candidates declined screening. Of the 441 SMA carrier spouse candidates, 13 were identified as SMA carriers. According to the screening test results for female spouse candidates of carrier males, the carrier prevalence among women was 2.95% (13/441). The confirmation test was performed with MLPA. MLPA testing could not be performed on five couples because they moved out of town or gave up on the marriage. In one couple, MLPA could not be confirmed only in the male spouse. Therefore, seven couples were identified as SMA carriers (7/441, 1.59%).

Additional findings indicate that two families opted for PGT and are currently undergoing IVF treatment. One family achieved a natural pregnancy resulting in a healthy baby, and their second pregnancy is ongoing with prenatal testing planned. Another family’s second pregnancy resulted in the birth of a healthy baby following a previous spontaneous miscarriage, with conception occurring naturally. In one case, a family conceived naturally, but prenatal testing revealed an SMA-affected fetus, which led to termination. Finally, two families have indicated that they do not plan to have children.

## 4 Discussion

### 4.1 Participation rates in premarital SMA screening

In this study, the aim was to determine the uptake of premarital SMA carrier screening and the frequency of SMA carriers in Samsun, Türkiye. In Samsun from 27 December 2021 to 31 May 2024, 19,988 people were screened. In 2022 and 2023, 18,198 marriages were performed, with a total uptake rate of 87.9% (16,004/19198). Broad-scale public screening for SMA carriers of reproductive age in the USA was identified to have an uptake of 98.7% (14). A meta-analysis including 169,000 people from 14 different studies found that the uptake for prenatal SMA carrier screening was 87–95% in the non-Black population, while it fell to 71% in the Black population (15). Since sociodemographic information for all screening participants was not available, we were unable to evaluate the factors influencing screening participation. According to meta-analysis results from Australia, there is a strong socioeconomic tendency toward participation in premarital carrier screening. People living in socioeconomically advantaged postcodes were shown to have a significantly higher likelihood of participating in premarital carrier screening compared to those living in more disadvantaged regions (16). Our study showed that participation increased over time with the training of health personnel and family physicians at the beginning of screening and public awareness activities. The role of trained health personnel in the success of the screening program is vital. For instance, Caner et al. recently highlighted that safety behavior and compliance with standard operating procedures in genetic laboratories in Istanbul are significantly predicted by training frequency and perceived institutional support (17). Another study conducted in our country showed that there is a significant lack of knowledge about the premarital SMA screening program among healthcare professionals. It was stated that re-evaluating professional training processes, promoting screening practices more effectively, and strengthening continuous education for healthcare workers would contribute positively to the implementation of screening programs (18). In this study, the identified uptake appears similar to that reported in the literature. These results emphasize the need to consider factors like sociocultural level, place of residence, educational level, and income level among the obstacles that may emerge for screening participation. Considering the importance of including the whole population in screening, our results indicate the need to work more carefully with the target population.

### 4.2 SMA carrier frequency

In this study, the carrier frequency was 1:44. A study of 3,049 subjects of reproductive age in China identified the SMA carrier prevalence as 1:49 (2.03%). In this study, the carrier prevalences were 2.3% in women and 1.9% in men, though the difference was not statistically significant (19). A study including 5,200 pregnant cases screened with a real-time fluorescence quantitative PCR method in China found that the carrier prevalence was 1.44% (95% confidence interval 1.31–1.65%) (20). Another study of 13,069 pregnant cases reported a carrier prevalence of 1:56 (21). In a study including Asians, differences in SMA carrier rates were observed among various ethnic origins. The frequency increased to 4.3% in the Tujia ethnic group (4). According to a meta-analysis from Australia, the SMA carrier rate was determined as 2% (16). In the Korean population, the SMA carrier frequency was identified as 1.8% (22). In this study, screening in women was only performed when a male partner was detected as a carrier. The carrier prevalence in screened female partners was higher than in men. The high prevalence of female carriers may introduce a selection bias and lead to misinterpretation. Factors such as consanguineous marrying within the same region, having similar genetic backgrounds, and the possibility of being related may have contributed to our female cohort representing a higher-risk group. In Saudi Arabia, a carrier screening study included 4,198 participants, with 45% women, and found the carrier prevalence in women (3.7%) was twice that in men (1.6%). The mean prevalence of SMA carriers (2.6%) was higher compared to other studies. When carriers were questioned about consanguineous marriage, nearly 27% were born from first-degree cousin marriages (23). In Asian countries where consanguineous marriage is common, the SMA prevalence is very high (24–27). A study using the MLPA method in Türkiye identified that the consanguineous marriage rate was 33% in cases with homozygous deletion (28). According to the 2018 Turkey Demographic and Health Survey, the rate of consanguineous marriages in Türkiye is 24% (29). This situation also supports the relatively high carrier prevalence detected in Turkey. In addition, consanguineous marriage which remains common in Türkiye may contribute to the relatively high carrier prevalence identified in this study. This hypothesis is essentially an interpretation supported by information reported in the literature. Nevertheless, although consanguinity data were not available for all couples, among the seven carrier couples for whom information could be obtained, 6 of the 14 individuals (42.8%) reported consanguineous marriage. A study conducted in Türkiye reported that, when examined by region, the rate of consanguineous marriage was 18% in the North, increasing to 33% in Southern Anatolia and 39% in Eastern Anatolia. In these two regions, first-degree consanguineous marriages accounted for a larger proportion of unions, whereas in other regions, second-degree and more distant consanguineous marriages were more prevalent. In Türkiye, cultural practices such as consanguineous marriage remain common, especially in rural areas, which can increase autosomal recessive conditions and cause regional differences in carrier frequencies. Beyond consanguinity, internal migration trends and the regional clustering of extended families in Türkiye could further affect variations in carrier frequencies. In some areas, large family networks often live in the same geographic locations across multiple generations, increasing the likelihood of shared ancestral backgrounds and, as a result, higher local carrier rates. Furthermore, cultural norms that influence premarital health-seeking behaviors, such as varying levels of trust in genetic testing and differing views on the necessity of screening, may determine who participates in screening programs and, in turn, affect regional prevalence rates (29, 30).

### 4.3 Early diagnosis, screening programs and global recommendations for SMA

Along with developments in SMA treatment, the importance of neonatal and premarital carrier screening emerged. The national genetic carrier screening program about reproductive health, beginning in 2013 in Israel, reduced the probability of impact from SMA disease by 57% (31). Considering that approximately 130–180 new SMA cases are expected annually in our country, it is expected that the national screening program will provide a certain reduction in the incidence of SMA in the coming years. Cases identified through NBS have been compared with patients diagnosed clinically after the onset of symptoms. Considering improvements in lifetime health outcomes and reductions in healthcare costs, the resulting cost savings demonstrate that NBS is cost-effective (32–34). In our country, both SMA NBS and premarital screening have been integrated into routine screening programs. This allows NBS to identify a potentially SMA-affected infant in cases where premarital screening is declined or yields a false-negative result. Thus, treatment can be initiated during the presymptomatic period, thereby preventing the disease’s negative economic burden. These two screening programs complement each other. The American College of Medical Genetics and Genomics (ACMG) recommends population-wide SMA screening and emphasizes that carrier screening does not replace NBS. Positive results from prenatal screening may cause increased stress, and the ACMG highlights that preconception screening is more strongly recommended than prenatal testing, as it provides couples with actionable information for reproductive decision-making (35). Health economic evaluations in rare diseases have gained importance in recent years. Studies conducted in European countries such as the United Kingdom, Italy, Netherlands, Belgium, and Portugal generally support that NBS is cost-effective for SMA (36). Since SMA is a rare disease, routinely published guidelines are limited, and different data sources exist across countries. Carrier screening programs also vary considerably across countries in scope, target populations, and whether costs are covered by the government (37). The Expert Consensus on Genetic Diagnosis of SMA published in China in 2020 recommended implementing SMA carrier screening in the general population (38). SMA is recommended for population screening because it is a relatively common, serious condition that often presents without a family history. A practical approach is to test one partner initially and proceed with testing the other only if the first is found to be a carrier. If the pregnancy has already begun, testing both partners simultaneously is advisable to ensure faster turnaround of results (39). No studies show the efficacy of screening performed in Türkiye. The data obtained will be beneficial in monitoring rare diseases in the future and in evaluating available programs. It is necessary to develop molecular methodologies for screening for rare diseases that may be added to the screening panel and to perform studies to assess the efficacy of screening. Obtaining more comprehensive epidemiological data about this topic in our country is important.

### 4.4 Detection of carrier couples and PGT results

During the study period, seven carrier couples were identified through screening. Two of them opted for PGT. When both parents are SMA carriers, the risk of birthing an infant with SMA disease is 25% for each pregnancy. Of children born to these couples, 50% are carriers without disease symptoms, while 25% are born healthy and do not carry the disease gene (40). Implementing screening in all family health centers ensures accessibility for the population which is a crucial factor for the success of the program. Optimization of accessibility should be ensured by cooperation with health professionals. Additionally, it is important to raise public awareness of the efficacy of carrier screening. The preimplantation genetic test (PGT) is implemented in two centers in Samsun. The common use of PGT will contribute to reducing the incidence of various autosomal recessive diseases. Due to the inherent structure of the screening test, a baby affected by SMA may be born as a result of this false reassurance. Prompt diagnosis and follow-up strategies to mitigate the impact of undetected carriers should not be neglected. Prenatal testing and NBS serve as complementary tools in managing this situation.

### 4.5 Future perspectives

In Türkiye, premarital carrier screening is performed for SMA and hemoglobinopathy disease. In Türkiye, a country with a high fertility rate among OECD countries, we believe expanding the carrier screening panel is a need that requires consideration due to the high incidence of consanguineous marriage. The target of genetic screening is to allow people to access the most accurate information at the right time to ensure they make informed decisions. In recent years, numerous studies worldwide have examined public perceptions and participation in carrier screening programs. In a French study, 19% of participants declined testing due to ethical or moral beliefs. Additionally, significant knowledge gaps regarding the tests were observed among both healthcare providers and the general public (41). Carrier screening programs for rare diseases require multidisciplinary collaboration. As a result, the literature on such programs is limited, and only representative examples of related government documents are accessible. Countries may benefit from identifying the most effective carrier screening techniques tailored to their expected disease prevalence. Screening programs should be developed in accordance with national health policies and should include cost analyses to evaluate potential benefits. By aligning public health priorities with genetic infrastructure, diseases and conditions that can be detected through carrier screening-and that may result in productivity loss, disability, mortality, or economically burdensome outcomes-can be prioritized. Integrating these screenings into routine programs with appropriate planning could serve as a model for other countries (37).

### 4.6 Limitations

Sociodemographic data for all screening participants were not available, limiting our ability to perform stratified analyses beyond basic coverage and carrier rate comparisons. Some information, such as age and residence, was available only for individuals who tested positive, preventing the application of logistic regression or other predictive analyses for screening participation. Future waves should include education, income, and ethnicity to identify participation barriers. Another limitation is that the qPCR test has 95% sensitivity and specificity for copy number variations on the 7th exon of the SMN1 gene; in reality, the carrier prevalence may be higher. Notable pitfalls of this study comprise the inherent false-negative risk associated with qPCR and the failure to obtain MLPA confirmation in some participants. Our study presents the results from Samsun province. However, cultural patterns and consanguineous marriage rates are thought to be similar in Northern region of Türkiye, these findings cannot be generalized to other provinces.

## 5 Conclusion

This study demonstrates that copy number variations in exon 7 of the SMN1 gene are not uncommon in the Turkish population, with a carrier prevalence of 2.26%. The observed uptake rate of 87.9% indicates promising public engagement; however, further strategies are needed to reach under-screened groups. Early and accessible genetic counseling services remain crucial, not only for preventing the birth of affected infants but also for fostering informed reproductive decision-making. The outcomes of this study provide valuable insights into the early implementation of a nationwide screening policy and highlight key areas—such as community outreach and counseling equity—for future program optimization. In the future, strengthening genetic counseling capacity and further expanding carrier panels will not only broaden the scope of clinical applications but also significantly improve the reproductive planning support provided to couples. As current national data on SMA prevalence and its geographic distribution remain limited, this study contributes a regional perspective that may inform both national policy development and future genetic screening research in Türkiye.

## Data Availability

The raw data supporting the conclusions of this article will be made available by the authors, without undue reservation.
