# Correction: From policy to practice: premarital spinal muscular atrophy screening as a public health initiative in northern Türkiye

**DOI:** 10.3389/fpubh.2026.1882048

**Published:** 2026-05-27

**Authors:** Feyza Nur Topcu Yenercag, Sule Ozturk, Gunay Kaya Tarhan

**Affiliations:** 1 Department of Public Health, Faculty of Medicine, Samsun University, Samsun, Türkiye; 2 Samsun Provincial Health Directorate, Samsun, Türkiye

**Keywords:** carrier screening, epidemiology, genetic counseling, premarital screening, spinal muscular atrophy (SMA)

The **Ethics statement** was erroneously given as:

“The study was approved by the SAMSUN University Clinical Ethics Board, 2024/11/8. The studies were conducted in accordance with the local legislation and institutional requirements. The data included in the study were obtained from individuals who had previously provided written informed consent for data collection and review. As the study was conducted retrospectively and was based on the review of existing records, no additional verbal or written informed consent was obtained from the participants.”

The correct **Ethics statement** is:

“The study was approved by the Samsun University Clinical Ethics Board (Decision No: GOKAEK 2024/11/8, Date: 05 June 2024). In addition, official institutional approval for conducting the study and use of the data was obtained from the Samsun Provincial Health Directorate Scientific Research Evaluation Commission (Date: 12 July 2024, Number: 248084573). The studies were conducted in accordance with the local legislation and institutional requirements. The data included in the study were obtained from individuals who had previously provided written informed consent for data collection and review. As the study was conducted retrospectively and was based on the review of existing records, no additional verbal or written informed consent was obtained from the participants.”

The original version of this article has been updated.

