# Correction: The impact of spatial normalization strategies on the temporal features of the resting-state functional MRI: spatial normalization before rs-fMRI features calculation may reduce the reliability

**DOI:** 10.3389/fnins.2026.1910411

**Published:** 2026-08-05

**Authors:** Zhao Qing, Xin Zhang, Meiping Ye, Sichu Wu, Xin Wang, Zuzana Nedelska, Jakub Hort, Bin Zhu, Bing Zhang

**Affiliations:** 1 Department of Radiology, The Affiliated Drum Tower Hospital of Nanjing University Medical School, Nanjing, China; 2 Institute for Brain Sciences, Nanjing University, Nanjing, China; 3 International Clinical Research Center, St. Anne's University Hospital Brno, Brno, Czechia; 4 Memory Clinic, Department of Neurology, Second Faculty of Medicine Charles University and Motol University Hospital, Prague, Czechia

**Keywords:** spatial normalization, resting-state fMRI, fMRI methods, reliability, fMRI preprocessing

There was a mistake in Figure 4 as published. During figure assembly, the whole-brain ICC map of the FC metric in Figure 4 was inadvertently replaced by an incorrect image. The histograms and quantitative analyses shown in the figure were generated from the correct FC data and are unaffected. The error only involved the visualization of the FC whole-brain ICC map.

The corrected Figure 4 appears below.

**Figure 4:**
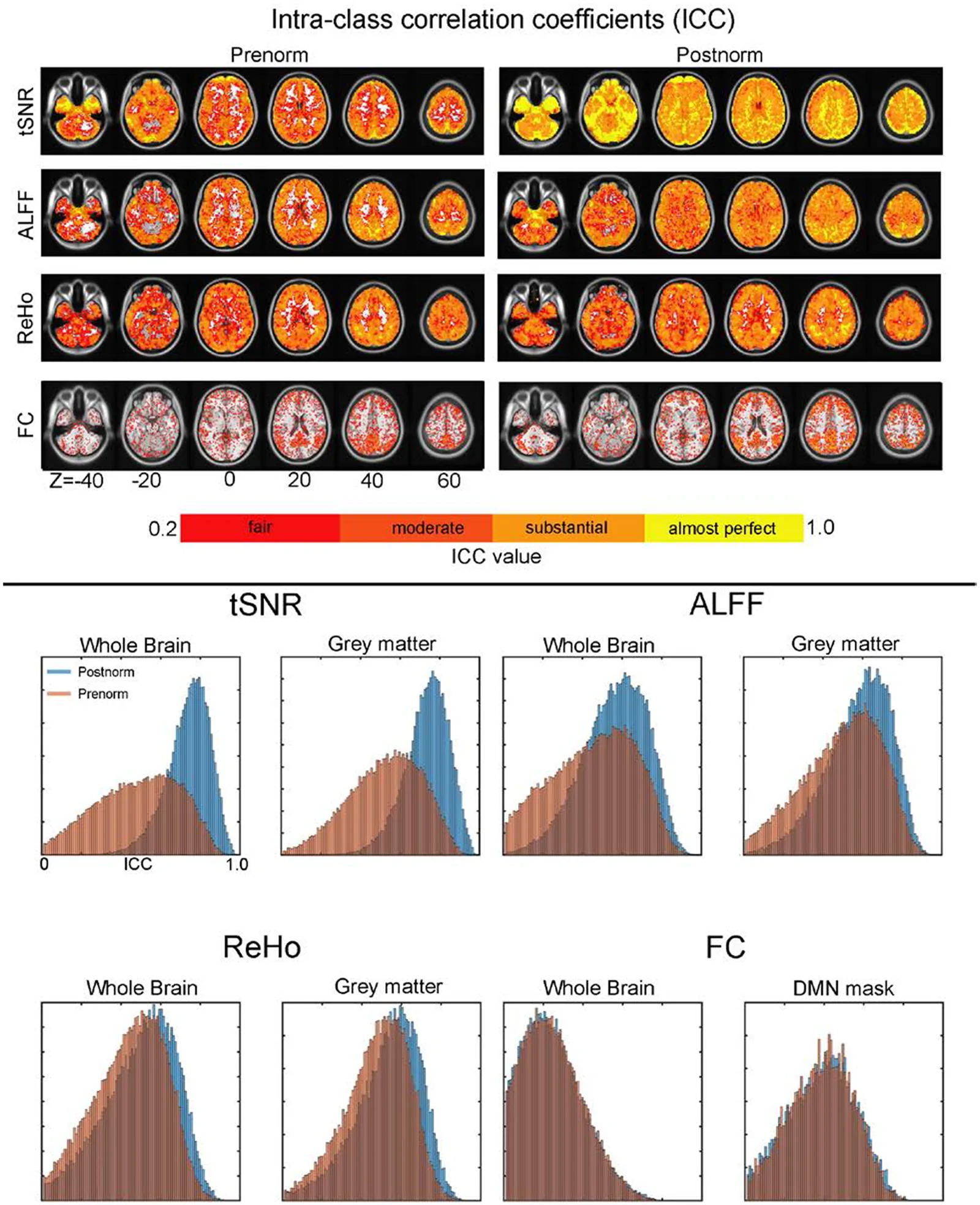
Effect of normalization strategies on intraclass correlation coefficients (ICCs) of the amplitude of low-frequency fluctuation (ALFF), regional homogeneity (ReHo), and functional connectivity (FC). **(Upper)** The left column showed the ICC values in each voxel after Prenorm and the right column for Postnorm. Each row for temporal signal-to-noise ratio (tSNR), ALFF, ReHo, and FC, respectively. The “fair reliability” mean means 0.2 < ICC < 0.4, “moderate” means 0.4 < ICC < 0.6, “substantial” means 0.6 < ICC < 0.8, and “almost perfect” means ICC > 0.8. **(Bottom)** Histogram of ICC. For each rs-fMRI index, the histograms of ICC for both normalization strategies were shown. For tSNR, ALFF, and ReHo, histograms within whole brain and gray matter regions were illustrated. For FC, besides whole brain, we focused on default mode network (DMN) regions to exclude regions with the very small FC values, as well as the controversial negative FC regions. According to a paired *t*-test, ICC of tSNR, ALFF, and ReHo is significantly higher when Postnorm is used compared to those when Prenorm is used. In contrast, FC showed no significant difference.

The original version of this article has been updated.

## Generative AI statement

Any alternative text (alt text) provided alongside figures in this article has been generated by Frontiers with the support of artificial intelligence and reasonable efforts have been made to ensure accuracy, including review by the authors wherever possible. If you identify any issues, please contact us.

